# Pulmonary Septic Emboli due to Azygos Vein Septic Thrombosis

**DOI:** 10.1155/2013/904057

**Published:** 2013-03-18

**Authors:** Ginius Pradhan, Khaldoon Shaheen, Mary Muoneke, Basel Altaqi

**Affiliations:** ^1^Internal Medicine Department, Case Western Reserve University/St. Vincent Charity Medical Center, Cleveland, OH 44115, USA; ^2^Cleveland Clinic Lerner College of Medicine of Case Western Reserve University, Department of Hospital Medicine, Institute of Medicine, Cleveland Clinic, 9500 Euclid Avenue, A13, Cleveland, OH 44195, USA; ^3^Internal Medicine Department, Case Western Reserve University/St. Vincent Charity Medical Center, Cleveland, OH 44115, USA; ^4^Department of Pulmonary and Critical Care Medicine, Case Western Reserve University/St. Vincent Charity Medical Center, Cleveland, OH 44115, USA

## Abstract

The triad of extrapulmonary infection, contiguous septic vein thrombosis, and septic pulmonary embolism is a rare complex but associated with significant morbidity and mortality. Septic azygos vein thrombosis is extremely rare and potentially serious since it may also cause pulmonary emboli and sudden death. We report a case of a 32-year-old woman with history of IV drug abuse who presented with epidural abscess and methicillin-resistant *S. aureus* (MRSA) bacteremia. Later she developed signs of septic pulmonary embolism secondary to septic azygos vein thrombosis. With early diagnosis, appropriate antimicrobial therapy, and control of the infectious source, resolution of the illness can be expected for most patients with avoidance of potential complications.

## 1. The Case

**Figure 1:**
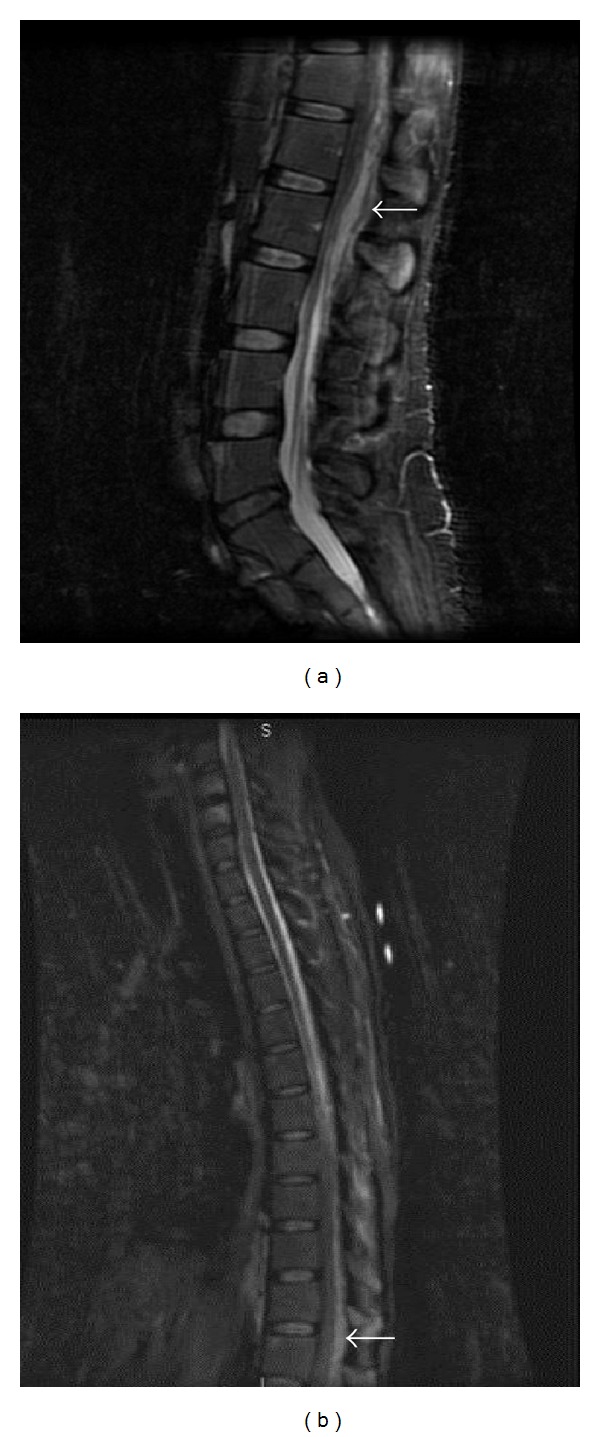
MRI of the lumbosacral spine (panel (a)) and thoracic spine (panel (b)), sagittal view: complex posterior epidural collection extending from lumbar L4 level cephalad into thoracic T7 associated central canal compromise and findings consistent with epidural abscess.

**Figure 2:**
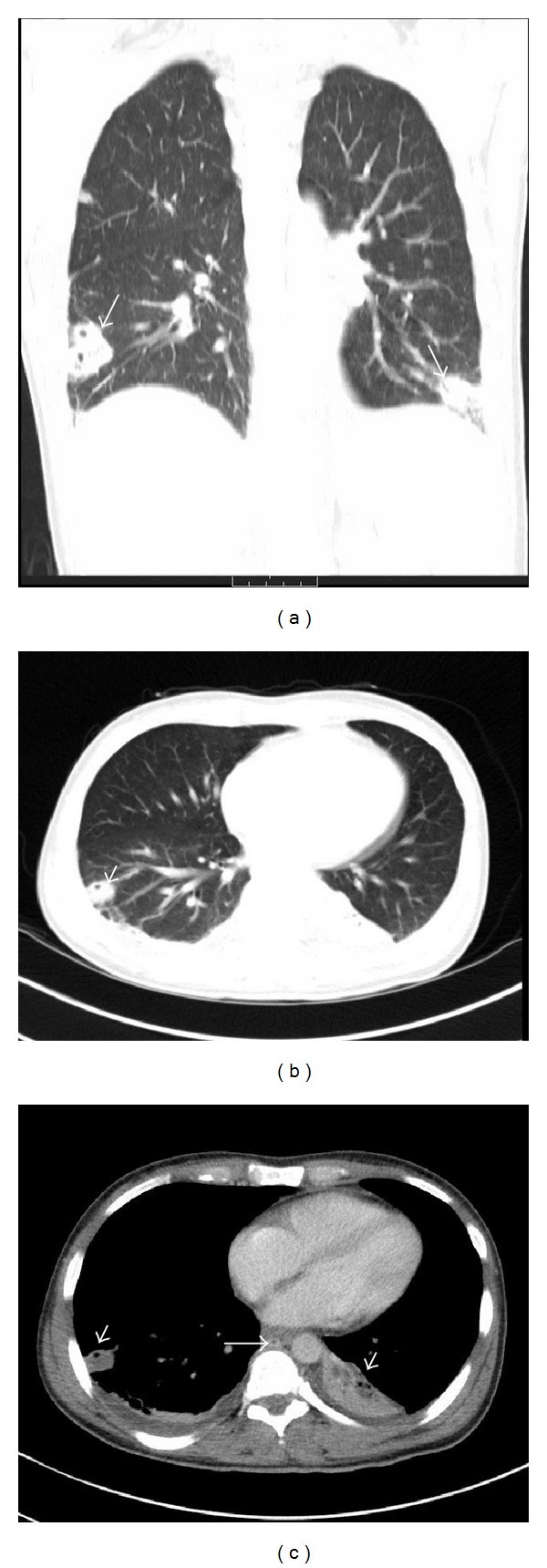
Computed tomography of the chest (panel (a): coronal view—lung window): multiple pulmonary cavitary lung lesions due to septic pulmonary emboli (arrows). Panel (b) (axial view—lung window) and panel (c) (axial view—mediastinal window) show multiple cavitary lung lesions due to septic pulmonary emboli (arrow heads). Filling defect is noted in the azygos vein suggestive of venous thrombus (arrow in panel (c)). There is associated mild pleural effusion bilaterally.

A 32-year-old woman presented to our emergency room reporting two-day history of progressively worsening back pain followed by weakness and numbness in both lower extremities. She denied any history of fever, chills, or night sweats. Her social history was significant for long history of IV heroin abuse. On examination, temperature was 36.8°C and normal other vital signs. BMI was 19. There were multiple needle marks in upper and lower extremities without abscesses or cellulitis changes. There was tenderness to palpation over the lumbosacral and thoracic spine. Strength was grade 4/5 in the lower extremities with brisk both knee reflexes. There was loss of sensation to touch from the umbilicus down. Blood workup showed WBC of 19 × 10^3^ cells/mm^3^ with bandemia (20%). Also was noted a high ESR of 101 mm/hr and CRP 121 mg/L. Urine toxicology was positive for opioids. Initial empiric antibiotic therapy was started on IV vancomycin and piperacillin/tazobactam for presumed vertebral infection secondary to intravenous drug abuse. Urgent MRI of thoracolumbar and thoracic spine showed a dorsally located epidural abscess extending from L4 up to T7 (Figure 1). She underwent emergent decompression surgery (laminectomy). The patient had remarkable improvement in her neurologic deficits postoperatively. On the 3rd postoperative day, the patient suddenly developed sudden onset cough, shortness of breath, and bilateral pleuritic chest pain. Contrast enhanced helical computed tomography of the chest showed multiple cavitary lung lesions consistent with septic pulmonary emboli (Figures 2(a)–2(c)). A filling defect was noted in the azygos vein consistent with venous thrombosis (Figure 2(c)). Venous duplex ultrasound of lower extremities did not reveal any evidence of deep venous thrombosis. Transesophageal echocardiography (TEE) did not show any vegetations. The diagnosis of septic deep vein thrombosis involving the azygos vein with secondary septic pulmonary emboli was made. Blood and abscess cultures grew methicillin-resistant *S. aureus (*MRSA), the antibiotics were tailored accordingly, she was continued on vancomycin, and later rifampin was added to her management. There was no anticoagulation therapy considered. The patient was discharged to continue the antibiotic therapy for additional eight weeks. Surveillance blood culture was negative and had improved follow-up imaging studies.

## 2. Discussion

Septic deep venous thrombosis (SDVT) or septic thrombophlebitis is rare but occasionally complicated by septic pulmonary embolism and associated with significant morbidity and mortality. The diagnosis is made based on clinical manifestations, culture data, and radiographic evidence of thrombosis. The triad of an active extrapulmonary source of infection, an adjacent venous thrombosis, and septic pulmonary emboli has been recently described in the literature, typically in the setting of deep soft tissue, bone, and vertebral infections. Historically, septic venous thrombosis and pulmonary embolism have been frequently associated with IV drug abuse, but recently due to a rise in using indwelling central catheters, infected intravascular devices are commoner causes [1]. Other predisposing factors include diabetes mellitus and immunosuppression. The most common causative organism is *Staphylococcus aureus* which plays a pivotal role in this complex due to a high tendency of *S. aureus* to promote venous thrombosis related to its intrinsic thrombogenic and proinflammatory potential [2].

Septic azygos vein thrombosis is potentially serious since it may also cause pulmonary emboli and sudden death. CT of the chest is the confirmatory imaging procedure of choice [3]. The commonly accepted principles of treatment for septic pulmonary embolism in the setting of septic venous thrombosis include prompt empiric administration of intravenous antibiotics, detecting and removing any potentially infected devices (e.g., intravenous catheters), and considering surgical intervention to remove purulent collections [4]. Although anticoagulation is the cornerstone of treatment for deep venous thrombosis, its use becomes controversial when the patient presents with septic venous thrombosis. Nevertheless, it may diminish the severity and duration of symptoms during the acute septic thrombotic event but may not directly facilitate thrombus dissolution [5]. However, patients with complications from septic vein embolism who fail to respond to antibiotic therapy alone may require anticoagulant therapy or inferior vena cava filter use. In this case, given her recent surgery, a decision of withholding anticoagulation was made and the patient improved significantly with intravenous antibiotics alone [3].

## 3. Conclusion

The key clinical point to remember in this case is how to establish the diagnosis of azygos vein septic thrombosis. Despite rarity, a particular high index of suspicion of azygos vein septic thrombosis is required in individuals who present with epidural or vertebral spine infection complicated by septic pulmonary emboli, particularly in those without evidence of cardiac source of embolism. Computed tomography of the chest is the confirmatory imaging procedure of choice.
